# Phylodynamic analysis and evaluation of the balance between anthropic and environmental factors affecting IBV spreading among Italian poultry farms

**DOI:** 10.1038/s41598-020-64477-4

**Published:** 2020-04-29

**Authors:** Giovanni Franzo, Claudia Maria Tucciarone, Ana Moreno, Matteo Legnardi, Paola Massi, Giovanni Tosi, Tiziana Trogu, Raffaella Ceruti, Patrizia Pesente, Giovanni Ortali, Luigi Gavazzi, Mattia Cecchinato

**Affiliations:** 1 Dipartimento di Medicina Animale, Produzioni e Salute (MAPS), Università di Padova, Legnaro, (PD) Italia; 2 Dipartimento di Virologia, Sezione di Brescia, Istituto Zooprofilattico Sperimentale della Lombardia e Emilia Romagna, Brescia, (BS) Italia; 3 Sezione di Forlì, Istituto Zooprofilattico Sperimentale della Lombardia e Emilia Romagna, Forlì Cesena, (FC) Italia; 4 Gesco sca, Cazzago San Martino, (BS) Italia; 5 Laboratorio Tre Valli, San Michele Extra, (VR) Italia

**Keywords:** Viral infection, Risk factors

## Abstract

Infectious bronchitis virus (IBV) control is mainly based on wide vaccine administration. Although effective, its efficacy is not absolute, the viral circulation is not prevented and some side effects cannot be denied. Despite this, the determinants of IBV epidemiology and the factors affecting its circulation are still largely unknown and poorly investigated. In the present study, 361 IBV QX (the most relevant field genotype in Italy) sequences were obtained between 2012 and 2016 from the two main Italian integrated poultry companies. Several biostatistical and bioinformatics approaches were used to reconstruct the history of the QX genotype in Italy and to assess the effect of different environmental, climatic and social factors on its spreading patterns. Moreover, two structured coalescent models were considered in order to investigate if an actual compartmentalization occurs between the two integrated poultry companies and the role of a third “ghost” deme, representative of minor industrial poultry companies and the rural sector. The obtained results suggest that the integration of the poultry companies is an effective barrier against IBV spreading, since the strains sampled from the two companies formed two essentially-independent clades. Remarkably, the only exceptions were represented by farms located in the high densely populated poultry area of Northern Italy. The inclusion of a third deme in the model revealed the likely role of other poultry companies and rural farms (particularly concentrated in Northern Italy) as sources of strain introduction into one of the major poultry companies, whose farms are mainly located in the high densely populated poultry area of Northern Italy. Accordingly, when the effect of different environmental and urban parameters on IBV geographic spreading was investigated, no factor seems to contribute to IBV dispersal velocity, being poultry population density the only exception. Finally, the different viral population pattern observed in the two companies over the same time period supports the pivotal role of management and control strategies on IBV epidemiology. Overall, the present study results stress the crucial relevance of human action rather than environmental factors, highlighting the direct benefits that could derive from improved management and organization of the poultry sector on a larger scale.

## Introduction

Infectious bronchitis virus (IBV) is currently classified in the species *Avian coronavirus*, genus *Gammacoronavirus*, family *Coronaviridae* (https://talk.ictvonline.org/). The viral genome is about 27Kb long and encodes different non-structural, accessory and structural proteins. Among those, the structural spike protein is by far the most extensively studied, because of its role in viral attachment, cell tropism and immunity^1^. Additionally, the current IBV classification in genotypes and lineages is based on the phylogenetic analysis of the corresponding genome region^2^.

IBV is one of the most relevant infectious diseases of poultry, causing remarkable economic losses due to respiratory and reproductive signs and increased mortality, especially when secondary infections occur or when nephropathogenic strains are involved^3^.

Currently, the most effective control measure is vaccine application, which proved to effectively reduce clinical signs emergence, infectious pressure and viral population size, at least when properly performed^4,5^. However, this approach cannot be considered a panacea, and some drawbacks cannot be denied. In fact, it must be kept in mind that IBV vaccination is not able to avoid animal infection and a prolonged circulation of field strains has been demonstrated even in vaccinated flocks^6^. Even when infectious bronchitis is properly controlled (i.e. asymptomatic infections), some economic losses have been reported in subclinically infected flocks^7^. Moreover, the co-circulation of field and live attenuated vaccines could enhance the likelihood of recombination and the vaccine induced immunity could promote and/or guide viral evolution^8,9^.

Biosecurity measures and the proper understanding of the factors influencing viral spread are thus of primary importance. The risk factors of virus introduction in a farm have been investigated in several studies, and include transport of live poultry, species and productive category, dominant winds^10,11^, sharing of personnel, fomites and means of transportation^12–16^. Other routes of transmission are summarized under the umbrella of ‘contiguous spread’. However, most of these studies are based on single outbreaks, during emergency scenarios (e.g. avian influenza outbreaks), and on the evaluation of factors associated to farm infection using a traditional statistical approach^17,18^. Therefore, a more comprehensive analysis of the ecological aspects behind the spread of IBV is largely lacking. Recently, advances in bioinformatics tools have allowed to link viral phylogeny and evolution to epidemiological factors, modeling the process of viral spreading over time, investigating its determinants and integrating landscape ecology with molecular epidemiology^19^. Therefore, a more accurate evaluation of IBV spreading determinants, informed on data collected over several years, could contribute in the understanding of this disease epidemiology.

One of the anthropic factors that affects and complicates the understanding of IBV epidemiology is the interaction between viral behavior and poultry production management. Modern poultry farming is typically featured by an integrated system, where all the production phases are organized and managed by a single entity/organization. The independence among the different integrated companies (i.e. separate hatcheries, means of transportation, feed delivery, slaughterhouses, etc.), even in the same country, should guarantee a certain protection from the introduction of new strains, at least from farms belonging to other companies.

Although theoretically plausible, this assumption has not been rigorously verified for IBV and the extent of potential breaches has never been quantified.

In the present study, the spreading process of the most relevant field genotype in Italy, IBV GI-19 lineage (previously known as QX), has been investigated using a continuous phylogeographic approach. The effect of different environmental and social variables, like altitude, road density, poultry population density, etc. on the spreading patterns has been also investigated using dedicated statistical tests. Finally, the migration of viral strains among integrated poultry companies was assessed and quantified.

## Results

### Dataset

**Figure 1:**
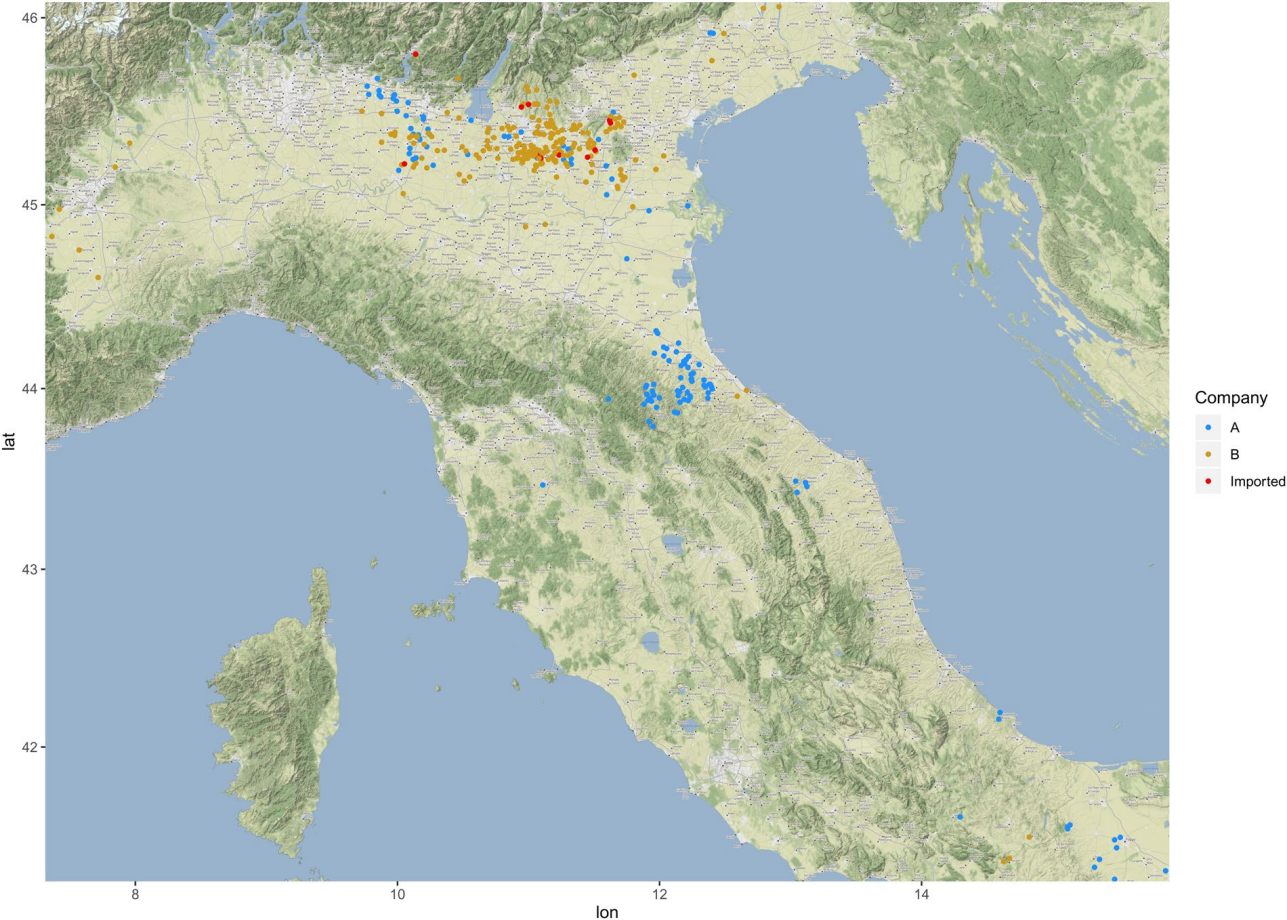
Location of farms from which samples have been obtained. Different companies have been color coded. Samples collected in *Company B* but clustering with *Company A* clade have been colored in red (herein named “Imported”). Farm location has been jittered using an internal routine of *ggplot* library to guarantee anonymity. The map was generated in R (version 3.4.4), using the library *ggmap*^51^.

A total of 361 QX sequences were included in the final dataset. Of those, 135 belonged to “Company A” and 226 to “Company B”. The sampled farm location is reported in Fig. 1. Overall, farms were mainly located in the “Pianura Padana” region (central area of Northern Italy) and, to a lesser extent, in North-Eastern, North-Western, Central and Southern Italy. Although the two companies tend to operate in different Italian regions, a clear overlapping was present in the high densely populated poultry area of Northern Italy (Fig. 1).

### QX-population genetics parameter estimation

All considered field sequences formed a monophyletic group including only Italian strains (Supplementary Fig. 1). TempEst investigation revealed that the positive correlation between genetic divergence and sampling time (i.e. R = 0.335) was suitable for phylogenetic molecular clock analysis^20^.

**Figure 2:**
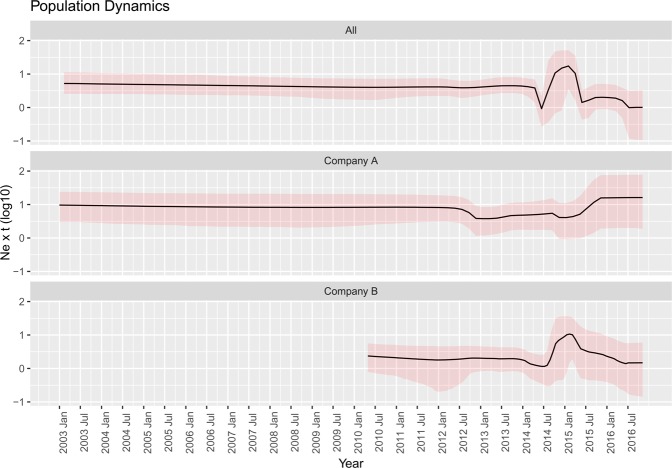
Mean relative genetic diversity (Ne x t) of the Italian GI-19 population over time. The results of the All Italian strains and of those sampled from (Company A and B) have been reported in different graphs. The upper and lower 95HPD values are reported as shaded areas.

The tMRCA of the overall QX population in Italy (i.e. QX genotype introduction) was estimated in 2003.52 [95HPD: 1999.73-2006.76] using the structured coalescent approach. Almost identical results were obtained including a third “*ghost” deme* (i.e. an estimated *deme* for which no sequences were available, representative of other unsampled companies and farms) in the analysis or using the “traditional” coalescent approach. When strains collected from integrated poultry companies were considered independently, the tMRCA was predicted in 2003.19 [95HPD: 1994.11-2010.3] for *Company A* and in 2010.6 [2007.26-2011.99] for *Company B*. The viral population dynamics evidenced a substantially constant Ne*t (Effective population size * generation time, or relative genetic diversity) with the remarkable exception of the period between mid-2014 and mid-2015, when a sudden fluctuation was observed. However, a quite different scenario was demonstrated between the two integrated poultry companies. In fact, *Company A* was featured by a substantially constant population size, with a minor decrease affecting particularly the period 2013-2015. However, the Ne*t 95HPD were relatively broad and at odds with the significance of the observed variations. On the contrary, a much more changeable pattern was observed in *Company B* (Fig. 2).

### Migration among companies

**Figure 3:**
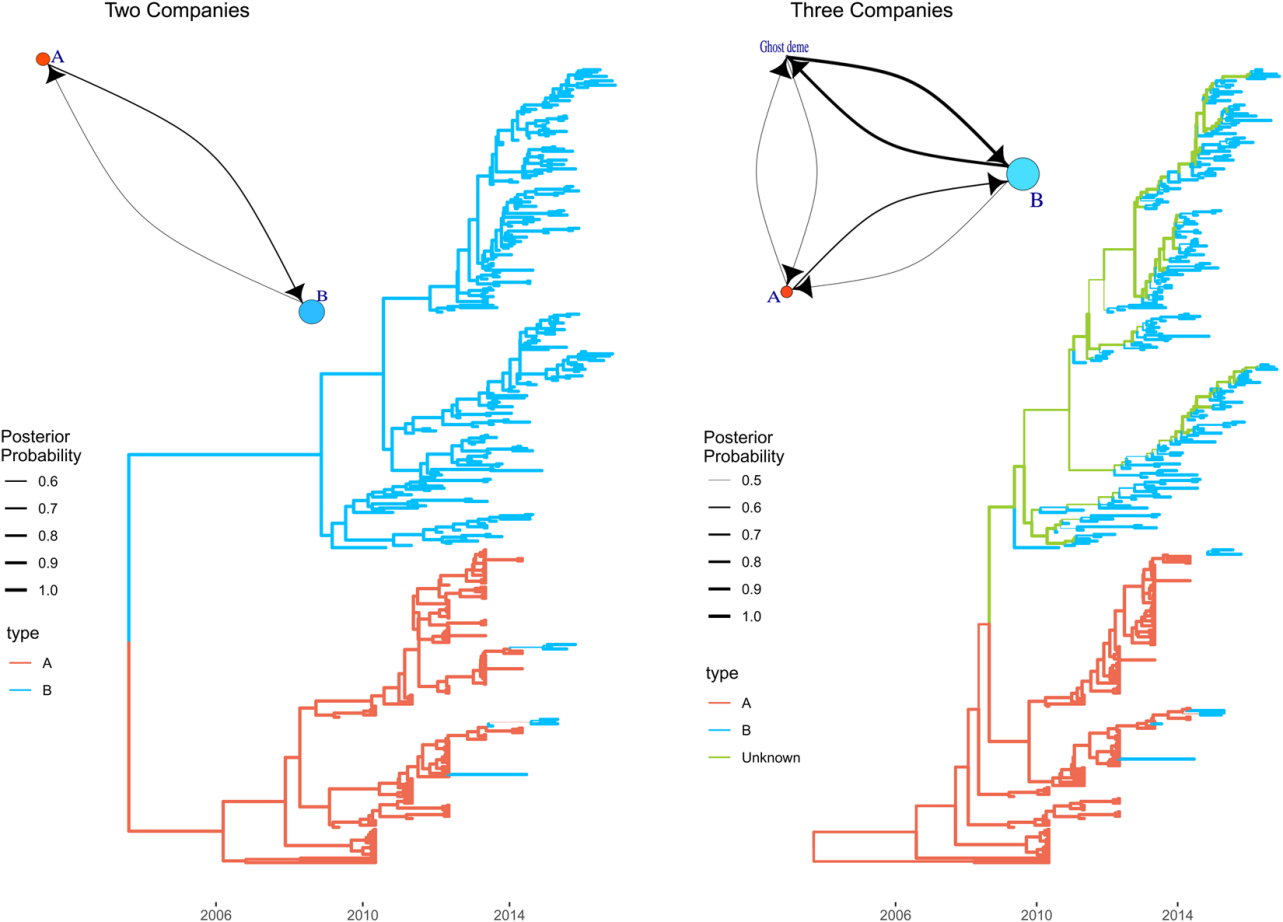
Structured coalescent-based phylogenetic tree of the samples included in the present study. Branch colors, as from legend, mark the inferred company where the ancestral strain was circulating, while branch width represents the posterior confidence of the inference. The trees reconstructed assuming just the *Company A* and *B demes* (left figure) and the one including also the *ghost deme* (right figure) are reported. In the top left insert it is reported the network depicting the migration rate between *Company A* and *Company B*. Arrows and circles size are proportional to the inferred migration rate and population size, respectively. Similarly, the top right insert reports the network of the migration rate between *Company A, Company B* and *ghost demes*.

The structured coalescent model fitted with the two company, evidenced the presence of 2 separate clades (Fig. 3A) for the 2 companies, with only 11 exceptions, represented by strains sampled in *Company B* but clustering in the *Company A* clade, thus suggesting the migration of strains from *Company A* to *Company B*. Accordingly, the migration rate from *Company B* to *Company A* was 2.5*10^−2^ [95HPD: 6.02*10^−2^-8.40*10^−2^], while the one from *Company A* to *Company B* was 6.66*10^−2^ [95HPD: 2.2*10^−2^-0.12]. The *demes* population size estimation suggested the viral population size of *Company B* being 1.86 times greater than the *Company A* (Fig. 3A).

When the third ghost *deme* was included in the analysis, a partially different scenario was observed (Fig. 3B).

*Company A* formed an independent clade (with the exception of the 11 strains sampled from *Company B*), evidencing transmission events occurring essentially within the same company.

On the other hand, several of the internal branches leading to strains collected from *Company B* were predicted to belong to the “ghost” *deme*, although not always with a high posterior probability (Fig. 3B). The viral spreading among farms of *Company B* appears to be at least partially meditated by other unsampled farms/companies (Fig. 3B). Even if the population size of the *ghost deme* was predicted to be 6.87 and 19.23 times smaller than the *Company A* and *Company B* ones, respectively, its role in the viral transmission was not negligible, being the estimated transmission rates:

from *Company B* to *Company A*: 1.56*10^−2^ [95HPD: 2.20*10^−7^-9.16*10^−2^]; from *ghost* to *Company A*: 1.27*10^−2^ [95HPD: 5.59*10^−8^-7.02*10^−2^]; from A to B: 5.68*10^−2^ [95HPD: 6.74*10^−4^-0.13]; from *ghost* to *Company B*: 1.58 [95HPD: 0.57- 2.73]; from *Company A* to *ghost*: 8.36*10^−2^ [95HPD: 1.64*10^−6^-0.43]; from *Company B* to *ghost*: 1.44 [95HPD: 4.04*10^−2^-3.51] (Fig. 3B).

### Phylogeographic analysis

**Figure 4:**
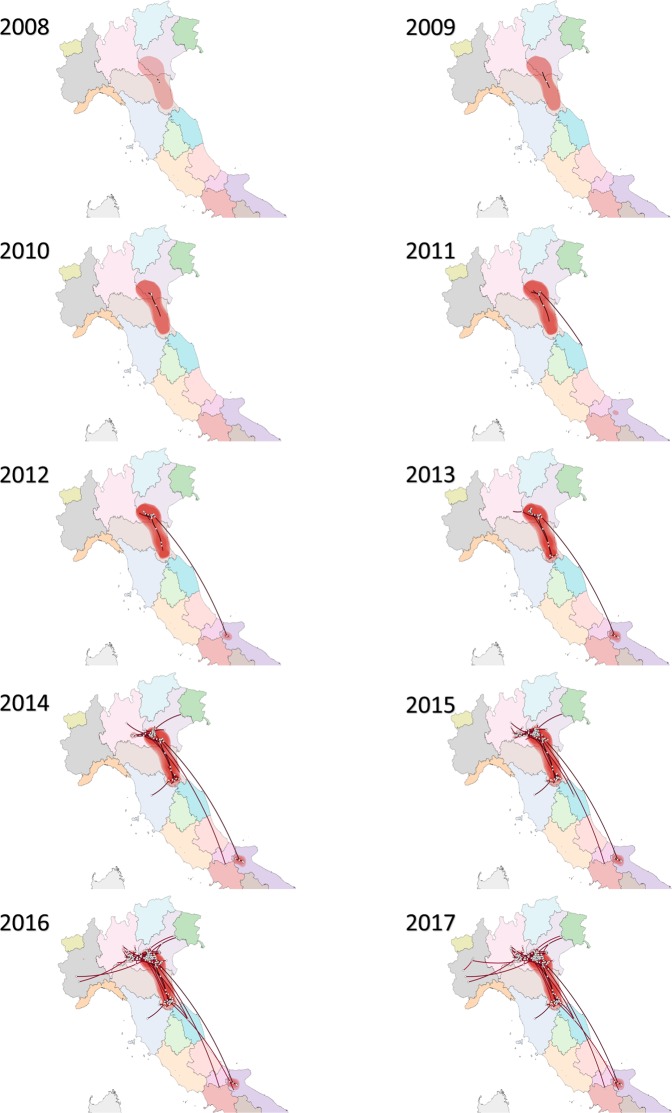
Results of phylogeographic analysis. The full posterior distribution of trees obtained in the continuous phylogeographic analysis is reported and the uncertainty (95HPD) on these estimates is reflected by contouring them with red polygons. Viral dispersal time has been represented by color coding the respective arrows from black (older) to red (newer).

**Figure 5:**
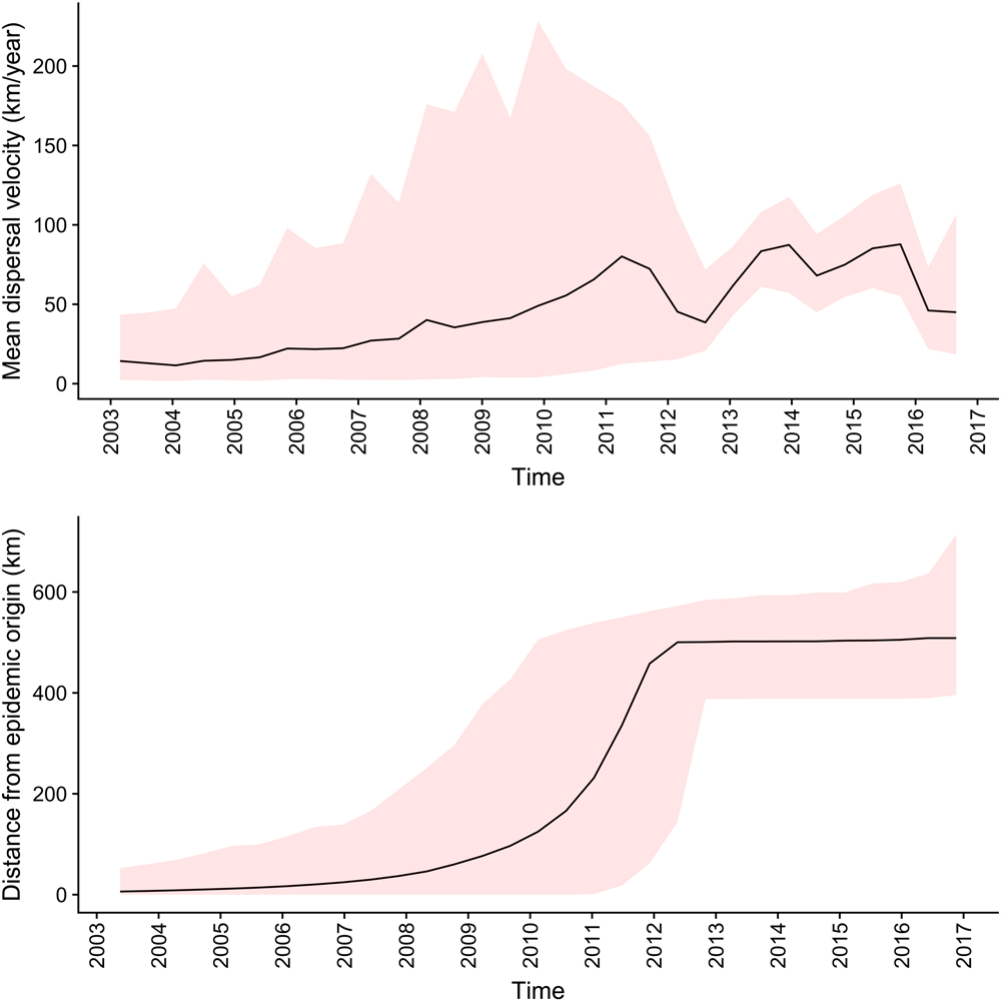
Estimated dispersion statistics of the QX epidemics. Upper figure: the mean dispersal velocity is reported over time. The red area corresponds to the 95% credible regions of the estimation. Lower figure: The geographic distance of the outbreaks wavefront from the estimated initial introduction is reported over time. The red area corresponds to the 95% credible regions of the estimated wavefront position.

All the samples phylogenetically belonging to *Company A* but collected from *Company B* originated from farms located in the high densely populated area of Northern Italy (Fig. 1). The continuous phylogeographic analysis reconstructed a spreading pattern originating from a single introduction in Emilia Romagna region (*Company A*), followed by a progressive expansion and persistence at high level in the Pianura Padana region. More rarely, spreading episodes toward other Italian regions were observed (Fig. 4). After QX introduction, the infection wave front increased slowly approximatively until 2009, when a rapid expansion led to the final distribution range by the middle of 2012 (Fig. 5). Accordingly, the dispersal velocity progressively increased in the first years after QX genotype introduction, peaking in the period 2009–2011 and then remaining essentially constant, despite some fluctuations (Fig. 5). The presence of a high dispersal velocity after 2012, when no further increase in wave front was observed, suggests that IBV continued to circulate at high rate after its first establishment in a region.

The analysis of the effect of different environmental factors on QX genotype dispersal velocity led essentially to negative results (i.e. absence of significant correlation). The only exception was represented by the poultry density SL model, which was positively and significantly correlated to viral dispersal velocity: D = 0.0225, percentage of D with p-value < 0.005 = 74%.

## Discussion

Despite the economic relevance, the epidemiology of IBV and the factors affecting its behavior have been only partially investigated. Even if a huge amount of knowledge and literature has accumulated over time, most of the reports are anecdotal or based on the analysis of single clinical outbreaks^17,21,22^. Although relevant pieces of information could be obtained, the risk of being biased by personal believes or the particular condition under investigation is high. A certain caution is thus required when inferring and extending the same conclusion on a broader/general scale. Moreover, most of the available studies are focused on Avian influenza and, to a lesser extent, Newcastle disease and Infectious laryngotracheitis^17,23,24^.

The aim of the present study was to construct an objective and statistically sound framework to understand IBV field strains behavior, the effect of control measures and the factors conditioning their epidemiology. The field of phylodynamics, and all related extensions, provides an invaluable tool for the study of viruses and particularly of rapidly evolving ones, whose evolution can be measured in “real time”, over the course of an epidemic^25^.

IBV QX genotype is the most relevant field strain in Italy^26,27^, and despite a relatively long circulation and the efforts devoted to its control, it still remains one of the main menaces for poultry industry profitability. Therefore, the understanding of the forces shaping its epidemiology would be of remarkable relevance in order to prevent the induced damages, rather than try to control them. Remarkably, the Italian IBV strains appear to originate from one introduction events only, as previously reported^27^. Therefore, it was possible to reconstruct IBV Italian strain evolution and epidemiological pattern without the biasing effect of strains recently introduced from other counties.

The implemented approach allowed to reconstruct the migration history of the QX genotype over time. The estimated introduction, in Emilia Romagna region, shortly predates the first detection, posing in favor of the effectiveness of the Italian monitoring and early detection systems. All the analyses, independently of the underlying statistical model, support that *Company A* was the first introduction site (Fig. 3). Thereafter, the virus circulation was limited to farms belonging to this company for years, until approximatively 2010, when *Company B* became involved. Contextually, a progressive increase in diffusion speed was noticed (Fig. 5), not unexpectedly considering the rising number of involved farms (especially at the border between Veneto and Lombardy regions, where most farms are located) and thus the increase in spreading potential and opportunity. The high farm density of this area has been described as a risk factor for different infectious diseases^22^, and IBV seems to be no exception. Interestingly, the viral population size remained relatively constant in this time period, evidencing that, even if QX strains were able to effectively spread from farm to farm, their replication was adequately controlled, likely by effective vaccination strategies. Actually, a certain slowdown in dispersal velocity was noticed in 2011-12, potentially because of a progressive decrease in naive populations availability. A dramatic change was observed in 2014, when a new spreading wave (Fig. 4) and an increase in diffusion rate (Fig. 5) and population size (Fig. 2) were detected. A more detailed analysis demonstrated that this variation affected Company B only (Fig. 2). A previous study has ascribed this episode to a change in the vaccination scheme adopted by this company, which moved from a heterologous Mass+793B based vaccination to a Mass only vaccination leading to an increased viral circulation and clinical outbreaks number^4^. Moreover, experimental studies demonstrated a significant reduction in R_0_ in vaccinated groups compared to unvaccinated ones^28^. It can therefore be speculated that the increase in infectious pressure within-farm and the higher flock susceptibility to infection could have enhanced the risk of IBV spreading to other farms and regions. In support of this hypothesis, the geographical spreading affected mainly Northern Italian farms (where *Company B* is located). Moreover, when a new double vaccination was implemented, the decrease in viral population size was mirrored by a reduction of dispersal velocity.

Continuous phylogeography showed that the areas interested by a more intensive viral circulation were those featured by a higher poultry density, and this evidence was confirmed by a statistically significant correlation between poultry density and dispersal velocity. The association between spatial proximity and farm infection is probably the most consistently reported risk factor for poultry infectious diseases^17,23,29^. Although an airborne transmission has been proposed for IBV, its occurrence has rarely been demonstrated experimentally^30^. However, the spatial proximity likely increases the likelihood of a greater number of horizontal contacts between farms, including the movement of people, vehicles and fomites between farms, as well as sharing similar risk factors (e.g. environmental conditions, climate, presence of wild animals, etc.)^16,23^. Based on these premises, the presence of segregated poultry companies should represent an effective obstacle to viral shedding and the obtained results partially confirm these evidence. The strains from different poultry companies formed two independent clusters, which suggests the effectiveness of independent production flow/chain in protecting farms from exogenous introductions. Additionally, the application of adequate biosecurity measures, enforced also by the Italian legislation, likely contributed in limiting new strain introduction.

The exceptions to this general rule were farms located in the high densely populated poultry area of Northern Italy, where an overlap between the two companies occurs. The unidirectionality of the viral flux from *Company A* to *Company B* implies that other factors, besides spatial proximity, must be in place. A detailed survey could shed some insights into relevant factors like different biosecurity measures, structural factors, vaccination strategy etc.. The mediation of other “actors” cannot also be excluded. In fact, the analysis of just two companies, however predominant they are on the Italian poultry sector, cannot be considered an accurate depiction of the Italian situation. Remarkably, the inclusion of a third *deme* (representative of other unsampled companies and farms) in the analysis model highlighted that several transmission events could be mediated by smaller entities operating in the same region. Actually, the high migration rate estimated between *Company B* and this *ghost deme* poses in favor of its pivotal role in maintaining an active IBV circulation.

Even if the idea of modeling *demes* for which no sequences are available could seem counterintuitive, previous studies showed that the structured coalescent can provide meaningful estimates even in absence of samples from one population^31^ and this approach has already been applied and proven effective for other diseases, including Ebola^32^. Since also Company A was evaluated in the same analysis run, the absence of relevant links between this company and the ghost *deme* further supports the analysis reliability, posing in favor of an actual interaction between *Company B* and the *ghost deme* rather than a mere low specificity of the method. A less effective control of IBV infection could be speculated for small companies, whose management capability and resources are limited compared to big-integrated companies. In fact, all Italian farms have to follow national legislation^33^ dictating the minimum biosecurity measures to be applied. However, integrated poultry farms, part of major companies, enforce additional managerial practices to increase biosecurity levels. Personnel and veterinarian formation, internal audits and periodic controls guarantee a higher level of application of the required standards, compared to most of small non-integrated farms.

The higher spatial overlap and the likely sharing of some infrastructures (e.g. streets, accessory personnel, services and infrastructures) could nevertheless have a negative indirect effect on the major companies, especially in Northern Italy where *Company B* is located. However, differences between *Company A* and *Company B* in the application of biosecurity measures and production flow management could also explain the different IBV epidemiology, as demonstrated by the dissimilar patterns in viral population fluctuations in the two companies (Fig. 2). A further risk factor that would deserve further investigation is the presence of the rural sector, which is highly concentrated in the densely populated poultry area of Northern Italy. This sector is characterized by a complex mix of growers, dealers and backyards flocks, often applying poor biosecurity measures and linked together by a poorly traceable contact network^34^. Although interactions with industrial poultry farming is hardly discouraged, illegal/indirect interactions have been documented and multiple epidemiological connections could result in a bidirectional transmission between the two sectors, as demonstrated in the Italian low pathogenicity Avian influenza (AI) outbreaks occurred in 2007–2009^34^. After these episodes, a stricter legislation has been developed, imposing limits to animal movements and more active surveillance in the rural sector. Nevertheless, no measures were taken for the monitoring and control of IBV in these enterprises, and therefore their role as sources of encroachment in intensive farming cannot be excluded.

Other environmental factors do not seem to play a relevant role in affecting viral dispersal. While climatic conditions like temperature, humidity and wind could actually affect viral viability and spreading, their effect could be circumvented by a transmission mediated by “fast-moving” vectors like trucks, personnel and, potentially, wild species^35,36^. More surprising could be the non-significant role of road density. However, it must be stressed that the available raster reported the overall density of roads, which could significantly differ from those preferentially used for live animal or their byproduct transportation, hindering the detection of an otherwise plausible risk factor. Therefore, the mapping of the live animal transportation pathways could provide remarkable benefits in IBV (and other infectious diseases) epidemiology understanding and control.

The present study demonstrates that IBV spreading potential is mainly affected by farm and poultry density overall, which can be reasonably claimed as a major risk factor. Other environmental/climatic variables do not seem to affect IBV epidemiology, stressing the pivotal role of human action and thus highlighting the direct benefits that could derive from an improved management and organization of the poultry sector on a larger scale. Actually, the integration of poultry production seems to provide a relevant constrain to IBV circulation, even though some differences were noted between the two considered companies. In fact, despite differences in management and applied control strategies likely playing a role, the presence in the same area of other minor poultry companies seems to represent a major issue, probably due to the less effective infection control ascribable to the sometimes lower organization capability and resources of small enterprises. The present study results emphasize the need of an active sharing of sequences and related molecular epidemiology data originating from all the actors in poultry production, allowing a proper depiction of the viral exchange dynamics, based on actual data rather than estimations. The obtained information would represent a fundamental substrate for the implementation of effective and shared efforts for the infection control on a broad regional scale.

## Materials and Methods

### IBV strain sampling, diagnosis and sequencing

Samples were collected for routine diagnostic purpose in the period 2012-2016 from poultry flocks belonging to the two main poultry companies (here named *Company A* and *Company B*) operating in Italy, which account together for about 90% of Italian poultry production. Samples were obtained mainly from outbreaks of respiratory disease, following a standard protocol that enforced the collection of a pool of 10 tracheal swabs from randomly selected birds. For each sampling, collection date and farm localization were recorded. All considered samples had been performed in the context of routine diagnostic activity and no experimental treatments or additional assays were implemented during the study. Therefore, no ethical approval was required to use specimens collected for diagnostic purpose. Additionally, several samples from *Company A* were already sequenced using the same protocol and published in Franzo *et al*.^27^. When detailed information on sampling farm and time could be traced back, these samples were included in the study. The permission to use the collected samples for research purpose was obtained from each company.

Swab pools were resuspended in 2 ml of PBS and vortexed. Thereafter, RNA was extracted from 200 µl of the obtained eluate using the High Pure Viral RNA Kit (Roche Diagnostics, Monza, Italy) kit. Diagnosis was performed by amplification and Sanger sequencing of the hypervariable region of the S1 region using the primer pair described by Cavanagh *et al*.^37^. Obtained chromatograms quality was evaluated using FinchTV (http://www.geospiza.com) and consensus sequences were generated using CromasPro (CromasProVersion 1.5).

### Sequence dataset preparation

All obtained sequences plus the reference dataset provided by Valastro *et al*. (2016) were aligned using MAFFT^38^ and a phylogenetic tree was reconstructed using IQ-TREE^39^ selecting as the best substitution model the one with the lowest Akaike’s information criterion, calculated using Jmodeltest^40^. The strains clustering with the GI-19 lineage (previously known as QX genotype) were selected and further evaluated for the presence of recombination in the considered region using RDP4^41^ and GARD^42^: to limit the computational burden the sequences were clustered using a 99% identity threshold using CD-HIT^43^ and a single representative sequence for each cluster was selected. These sequences plus the Valastro *et al*. (2016) references were re-aligned and recombination analysis was performed. Recombinant sequences, including the ones belonging to the same cluster, were removed from the dataset. Finally, the dataset was re-expanded to the original size and sequences identical or closely related (p-distance <0.01) to the QX-based vaccines administered in Italy were also excluded. To evaluate the distribution of Italian GI-19 strains in the international scenario, an extensive dataset of S1 IBV sequences was downloaded from GenBank and a phylogenetic tree was reconstructed as previously described. To reduce computational complexity and increase interpretation easiness (without losing information), only one sequence representative of all identical ones was selected using CD-HIT and included in the analysis.

The presence of an adequate phylogenetic signal was assessed by a likelihood mapping analysis performed with IQ-TREE. TempEst was used to preliminarily evaluate the temporal signal of the Italian QX phylogeny and therefore the applicability of molecular clock-based methods^20^.

### Strain migration among integrated poultry companies

IBV QX strain migration among companies was evaluated using the structured coalescent-based approach implemented in the MultiTypeTree extension of BEAST2^44^. According to this model, the considered population is divided in a series of *demes*, which can be imagined as different islands, featured by their own populations size and interconnected by a certain migration rate among them.

In the particular Italian QX scenario, the serially sampled (i.e. with known collection date) strains were used to infer the migration rate and history between the two integrated poultry companies (i.e. considered as different *demes*) over time. Additionally, the Bayesian approach implemented in BEAST allowed to contextually estimate other population parameters, including the time to most recent common ancestor (tMRCA), evolutionary rate and population size.

Accounting for the presence of other farms and companies operating in the Italian poultry sector, which could take part in or mediate the viral transmission among the investigated major companies, a third *“ghost” deme* (a *deme* for which no sequences were available) was added to the model^31^. The priori of the *ghost deme* size was set to one tenth of the other *demes*, according to the estimated poultry population distribution. However, broad priori distribution (i.e. relatively uninformative priori) was chosen to avoid constrains or biases in the parameter posterior estimation.

For all analyses, the best substitution model (TN93 + G_4_) was selected based on the Bayesian information criterion, calculated using Jmodeltest^40^, while the relaxed lognormal molecular clock model was selected based on marginal likelihood calculation and comparison using the Path Sampling and Stepping Stone method^45^.

The final estimations were obtained performing a 200 million generation Markov chain Monte Carlo run, sampling parameters and trees every twenty thousand generations. Results were visually inspected using Tracer 1.5 and accepted only if mixing and convergence were adequate and the Estimated Sample Size was greater than 200 for all parameters.

Parameter estimation was summarized in terms of mean and 95% Highest Posterior Density (HPD) after the exclusion of a burn-in equal to 20% of the run length. Maximum clade credibility (MCC) trees were constructed and annotated using Treeannotator (BEAST package).

Results consistency was also evaluated performing a “traditional” serial coalescent analysis in BEAST 1.8.4^46^. The same substitution and clock model of the structured coalescent analysis were selected, while a nonparametric skyline population model was chosen to reconstruct the viral population dynamic over time^47^. Independent analysis for each integrated company were also performed using the same approach but generating two new datasets including only the sequences collected from a specific company. However, sequences introduced from one company to the other were excluded from the company-specific analysis since they did not share a common evolution history.

### Continuous phylogeography and determinants of IBV spreading

The history of QX dispersal was reconstructed over time using the continuous phyogeographic approach described by Lemey et al.,^48^ using BEAST 1.8.4. Substitution and clock models were selected as previously described. Similarly, the Gamma Relaxed Random Walk was preferred over the other phylogeographic continuous diffusion models based on the marginal likelihood calculation and comparison using the Path Sampling and Stepping Stone method^45,48^. The final estimations were obtained performing a 200 million generation Markov chain Monte Carlo run, sampling parameters and trees every twenty thousand generations. Results were visually inspected using Tracer 1.5 and accepted only if mixing and convergence were adequate and the Estimated Sample Size was greater than 200 for all parameters. The reconstruction of QX movements over time within Italian borders was obtained using SpreaD3, summarizing and visualizing the full posterior distribution of trees obtained in continuous phylogeographic analyses^49^.

Pattern and determinants of viral spreading were evaluated as described by (Dellicour *et al*.)^19^, using the *seraphim* R library^50^. The history of lineage dispersal was recovered from the posterior trees generated using BEAST and annotated with ancestral longitude and latitude reconstruction. Particularly, the distance, duration and velocity of spatial dispersal were recoded as vectors and used to generate different summary statistics of viral spreading, including dispersal velocity and maximal wave front distances (measured from the location of the tree root).

Several environmental/social variables were considered to determine if they were associated with the dispersal rate of IBV lineages. The environmental rasters describing the variables of are shown in Supplementary Fig. 2.

More in detail, the values in the raster (i.e. altitude, population density, poultry density, temperature, etc.) were used to associate a weight to the abovementioned vector. Two models of spatial movements were considered: (1) “straight line (SL) path” model, assuming a straight movement between the starting and ending locations of each branch (i.e. the branch weight is computed as the sum of raster cells through which the straight line passes); (2) “least cost (LC) path” model, using a least cost algorithm (i.e. the branch weight is computed as the sum of the values of cells transition values between adjacent cells along the least-cost path). In this model, the analyzed environmental variable can be considered both as a conductance (i.e. enhancing viral dispersal through the cells with higher values) or resistance factor (i.e. allowing an easier dispersal through cells with lower values). Both instances were evaluated for each considered factor.

The obtained “environmental” weights were used to calculate a regression with the branch duration and the corresponding coefficient of determination (R^2^_env_) was obtained. A null coefficient of determination (R^2^_null_) was also calculated assuming the null raster (i.e. when only the spatial distance of each movement is assumed to affect branch duration). The statistic D = R^2^_env_ – R^2^_null_ was selected as final outcome, and describes how much the regression is strengthened when the spatial variation in the environmental variable is included. To account for the phylogenetic uncertainness, the D statistic was calculated for each tree of the posterior distribution. However, for computational constraints, the number of posterior trees was down-sampled to 1000 after discharging a 20% burn-in. Only the environmental variables with more than 90% of D statistics > 0 were considered for further analysis. Particularly, the significance of D statistic of those variables was assessed against a D null distribution obtained by randomizing 1000 times the phylogenetic nodes location under the constraint that branch length remained equal. A p-value was generated for each initial tree, therefore a percentage of the trees with p-value < 0.05 could be calculated, which can be interpreted as a posterior probability of observing a significant correlation between lineage movements and considered environmental variable. According to Dellicour *et al*., (2016), a percentage of p-value < 0.05 greater than 50% was considered a strong evidence that the environmental variable is associated to viral movement speed^19^.

## Supplementary information

Supplementary figure 1.

Supplementary figure 2.
