# Supplementary material for: Phylodynamic analysis and evaluation of the balance between anthropic and environmental factors affecting IBV spreading among Italian poultry farms
Source: Sci Rep. 2020 Apr 29;10:7289. doi: 10.1038/s41598-020-64477-4 (PMC7190837; doi:10.1038/s41598-020-64477-4)
Supplement: Supplementary file 1 — Supplementary figure 1. [file 41598_2020_64477_MOESM1_ESM.pdf]

Phylodynamic analysis and evaluation of the balance between anthropic and environmental factors affecting IBV spreading among Italian poultry farms.

Giovanni Franzo[1*], Claudia Maria Tucciarone[1], Ana Moreno[2], Matteo Legnardi[1], Paola Massi[3], Giovanni Tosi[3], Tiziana Trogu[2], Raffaella Ceruti[4], Patrizia Pesente[5], Giovanni Ortali[5], Luigi Gavazzi[4], Mattia Cecchinato[1].

[1] *Dipartimento di Medicina Animale, Produzioni e Salute (MAPS), Università di Padova, Legnaro (PD), Italia.*

[2] *Dipartimento di Virologia, Sezione di Brescia, Istituto Zooprofilattico Sperimentale della Lombardia e Emilia Romagna, Brescia (BS), Italia.*

[3] *Sezione di Forlì, Istituto Zooprofilattico Sperimentale della Lombardia e Emilia Romagna, Forlì Cesena (FC), Italia.*

[4] *Gesco sca, Cazzago San Martino (BS), Italia.*

[5] *Laboratorio Tre Valli, San Michele Extra (VR), Italia.*

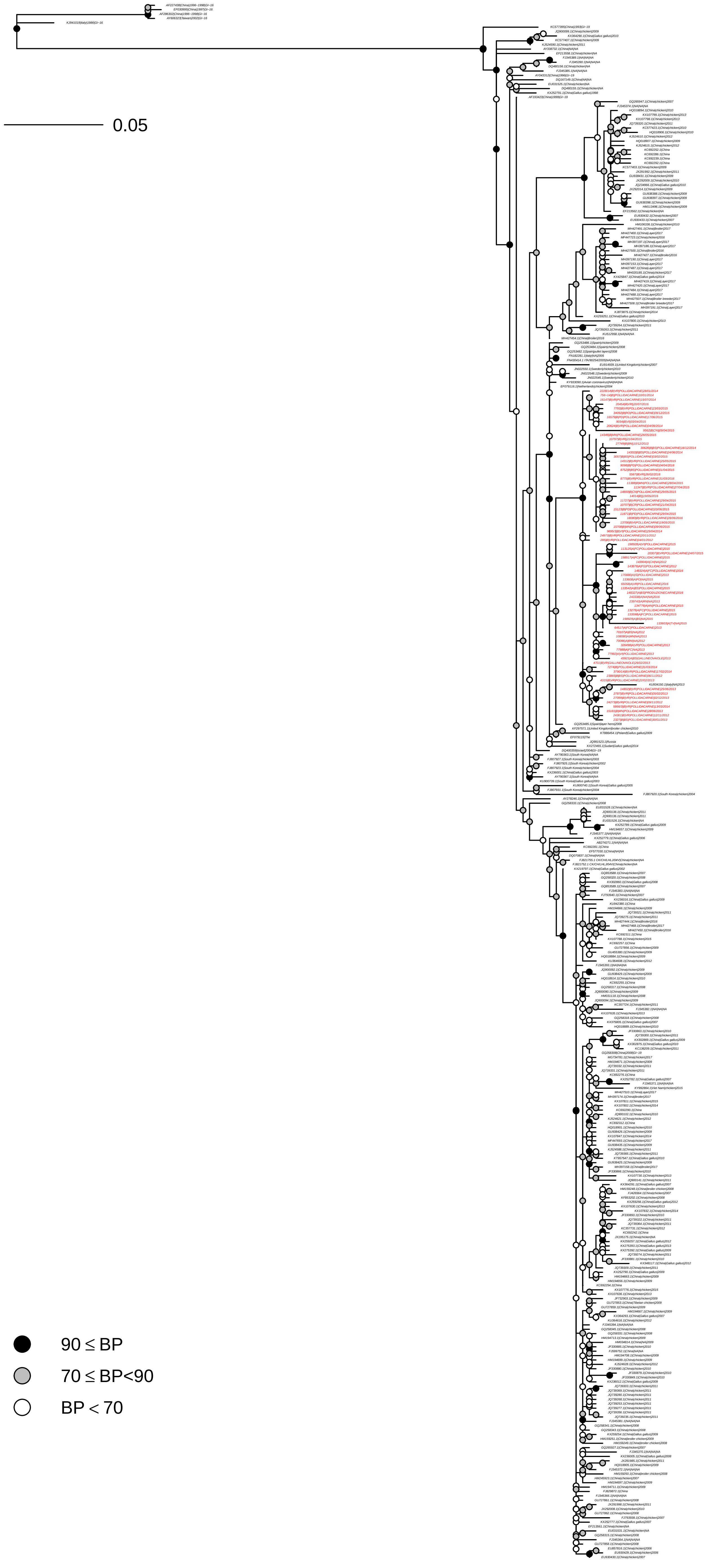

Supplementary figure 1. Maximum likelihood phylogenetic tree based on all available QX (GI−19) strains.
Nodes bootstrap support has been reported as color−coded circles. Italian strains included in the present study are highlightedin red.
For graphical reason, only one sequence representative of all identical ones has been included. The tree has been rooted using the GI−16 lineage.
It is possible to zoom in to appreciate further details and tip labels
