# Supplementary material for: Phylodynamic analysis and evaluation of the balance between anthropic and environmental factors affecting IBV spreading among Italian poultry farms
Source: Sci Rep. 2020 Apr 29;10:7289. doi: 10.1038/s41598-020-64477-4 (PMC7190837; doi:10.1038/s41598-020-64477-4)
Supplement: Supplementary file 2 — Supplementary figure 2. [file 41598_2020_64477_MOESM2_ESM.pdf]

Phylodynamic analysis and evaluation of the balance between anthropic and environmental factors affecting IBV spreading among Italian poultry farms.

Giovanni Franzo[1*], Claudia Maria Tucciarone[1], Ana Moreno[2], Matteo Legnardi[1], Paola Massi[3], Giovanni Tosi[3], Tiziana Trogu[2], Raffaella Ceruti[4], Patrizia Pesente[5], Giovanni Ortali[5], Luigi Gavazzi[4], Mattia Cecchinato[1].

[1] *Dipartimento di Medicina Animale, Produzioni e Salute (MAPS), Università di Padova, Legnaro (PD), Italia.*
[2] *Dipartimento di Virologia, Sezione di Brescia, Istituto Zooprofilattico Sperimentale della Lombardia e Emilia Romagna, Brescia (BS), Italia.*
[3] *Sezione di Forlì, Istituto Zooprofilattico Sperimentale della Lombardia e Emilia Romagna, Forlì Cesena (FC), Italia.*
[4] *Gesco sca, Cazzago San Martino (BS), Italia.*
[5] *Laboratorio Tre Valli, San Michele Extra (VR), Italia.*

Supplementary figure 2) Rasters of environmental variables used in the present study.

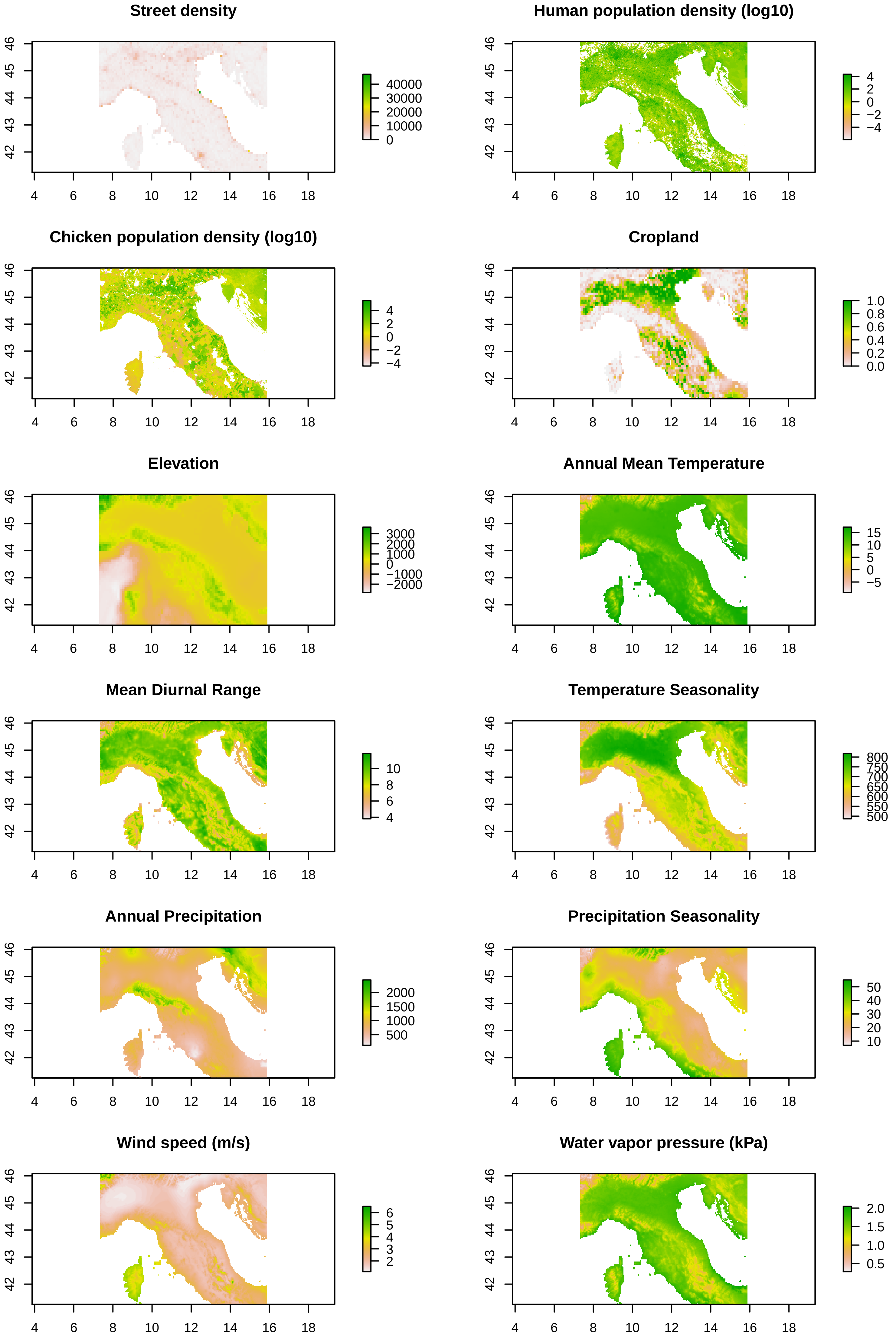
Street density
40000
30000
20000
10000
0
Human population density (log10)
4
2
0
−2
−4
Chicken population density (log10)
4
2
0
−2
−4
Cropland
1.0
0.8
0.6
0.4
0.2
0.0
Elevation
3000
2000
1000
0
−1000
−2000
Annual Mean Temperature
15
10
5
0
−5
Mean Diurnal Range
10
8
6
4
Temperature Seasonality
800
750
700
650
600
550
500
Annual Precipitation
2000
1500
1000
500
Precipitation Seasonality
50
40
30
20
10
Wind speed (m/s)
6
5
4
3
2
Water vapor pressure (kPa)
2.0
1.5
1.0
0.5
46
45
44
43
42
4
6
8
10
12
14
16
18
